# Optimal slice thickness for object detection with longitudinal partial volume effects in computed tomography [JACMP, 18(1) 251‐259, 2017]

**DOI:** 10.1002/acm2.12330

**Published:** 2018-04-19

**Authors:** Pascal Monnin, Nicolas Sfameni, Achille Gianoli, Sandrine Ding

**Affiliations:** ^1^ Haute Ecole de Sante Vaud (HESAV) Filière TRM University of Applied Sciences and Arts of Western Switzerland (HES‐SO) Lausanne Switzerland; ^2^ Institute of Radiation Physics (IRA) University Hospital of Lausanne (CHUV) Lausanne Switzerland

The authors would like to correct the correspondence information^1^ from “Haute Ecole de Santé Vaud (HESAV), Filière TRM, Lausanne, Switzerland” to “Haute Ecole de Santé Vaud (HESAV), Filière TRM, University of Applied Sciences and Arts of Western Switzerland (HES‐SO), Lausanne, Switzerland.”

## References

[1] Monnin P , Sfameni N , Gianoli A , Ding S . Optimal slice thickness for object detection with longitudinal partial volume effects in computed tomography. J Appl Clin Med Phys. 2017;18:251–259. 10.1002/acm2.12005 PMC5689876 28291920

